# Case report: First report of *Legionella pneumophila* and *Bordetella bronchiseptica* coinfection in an immunocompromised patient

**DOI:** 10.3389/fmed.2024.1470567

**Published:** 2024-10-22

**Authors:** Marilena La Sorda, Ivana Palucci, Daniele Natalini, Silvia Fillo, Francesco Giordani, Francesco Paglione, Anella Monte, Florigio Lista, Fabiola Mancini, Antonietta Girolamo, Maria Cristina Rota, Maria Grazia Caporali, Rosalba Ricci, Christophe Ginevra, Sophie Jarraud, Maurizio Sanguinetti, Maria Scaturro, Maria Luisa Ricci

**Affiliations:** ^1^Dipartimento di Scienze di Laboratorio e Infettivologiche, Fondazione Policlinico Universitario “A. Gemelli” IRCCS, Rome, Italy; ^2^Dipartimento di Scienze biotecnologiche di base, cliniche infettivologiche e peri-operatorie – Sezione di Microbiologia, Università Cattolica del Sacro Cuore, Rome, Italy; ^3^Dipartimento di Scienze dell'Emergenza, Anestesiologiche e della Rianimazione, Fondazione Policlinico Universitario “A. Gemelli” IRCCS, Rome, Italy; ^4^Defence Institute for Biomedical Sciences, Rome, Italy; ^5^Dipartimento di Malattie Infettive, Istituto Superiore di Sanità, Rome, Italy; ^6^National Reference Centre for Legionella, Hospices Civils de Lyon, Lyon, France; ^7^ESCMID Study Group for Legionella Infections (ESGLI), Basel, Switzerland

**Keywords:** legionnaires' disease, *Legionella pneumophila*, *Bordetella bronchiseptica*, coinfection, cgMLST

## Abstract

Legionnaires' disease (LD) is a serious type of pneumonia, typically contracted by susceptible people through the inhalation of aerosols contaminated with *Legionella pneumophila (Lp)*. In this report, the first case of coinfection with *Lp*–*Bordetella bronchiseptica (Bb)* is described. A possible source of the Lp infection may be the hotel in Paris (France) where the patient had stayed before developing the symptoms. The Bb infection may have been transmitted by the dog with which he had constant contact, although this has not been proven.

## Introduction

Legionnaires' disease (LD) is an infection caused by *Legionella pneumophila* (*Lp*), a Gram-negative waterborne pathogen noted by the World Health Organization as posing the highest health burden in the European Union (1). *Lp* infection is acquired through the inhalation of infectious aerosols originating from water systems in buildings, such as showers, fountains, spa pools, and cooling towers. LD cases can be acquired in the community, through travel, or in nosocomial settings. Male individuals aged 65 years and older, especially those with underlying diseases, alcohol abuse, smoking habits, or immunosuppression, are mainly susceptible (2). In Europe, Italy is one of the four countries, along with France, Germany, and Spain, responsible for 72% of all notified cases; the majority (82%) of the cases are travel-associated legionnaires' disease (TALD) cases (3). Few cases both in Europe (10%) and in Italy (0.8%) are diagnosed through the culture method. Consequently, since the strain is not available, it is difficult to trace the origin of the infection (3, 4).

*Bordetella* is a strictly aerobic, non-fermentative, catalase-positive, and oxidase-positive Gram-negative coccobacillus, consisting of 16 species. *Bordetella pertussis* (*Bp*), *Bordetella parapertussis* (*Bpp*), and *Bordetella bronchiseptica* (*Bb*) are defined as “Classical *Bordetellae*,” all of which cause respiratory infections ranging from severe to mild or asymptomatic (5, 6). However, while *Bp* and *Bpp* can infect only humans and cannot survive in the environment, *Bb* is the major causative agent of the canine infectious respiratory disease complex (7). Although *Bb* is seldom considered infectious for humans, transmission from the dog to the owner is possible (8). Sporadic cases of *Bb* infection have primarily been linked to debilitated or immunosuppressed patients (9, 10).

In this article, we present for the first time a coinfection with *Lp–Bb* in a 69-year-old immunocompromised man with chronic renal failure, hypertension, and IgG Lambda multiple myeloma (MM) diagnosed in 2014.

## Methods

### Case description

On 13 January 2023, a patient was admitted to the emergency room (ER) with fever and worsening dyspnea, which had started 5 days earlier. He reported having traveled to Paris for New Year's Eve and had previously undergone autologous hematopoietic cell stem transplantation with a positive outcome in 2015. Due to multiple recrudescences of the MM, he was actively receiving fourth-line chemotherapy with isatuximab plus pomalidomide and dexamethasone (IsaPd), the last dose of which was administrated 10 days before his travel to Paris (France).

During the first 2 days in the ER, he rapidly developed severe hypoxemic respiratory failure and hemodynamic instability. He was initially treated with non-invasive mechanical ventilation, followed by invasive mechanical ventilation, which required sedation, paralysis, and orotracheal intubation. While he was in the ER, vasopressors, such as norepinephrine, were administered to the patient, and empiric antibiotic therapy was initiated with three doses of intravenous (IV) clarithromycin (500 mg, twice a day) and one dose of Meropenem (500 mg).

From the beginning of the antibiotic therapy, the patient clinically improved, and we were able to extubate him 48 h after his admission to the intensive care unit (ICU). When the patient was experiencing spontaneous breathing, we initially supported oxygenation using high-flow oxygen therapy delivered by a nasal cannula but soon switched to conventional oxygen therapy. Norepinephrine was reduced and stopped during the first 24 h in the ICU. On 15 January, the patient was moved to the general intensive care unit (ICU); however, he remained deeply hemodynamically unstable and required a high-dose noradrenaline infusion and continuous renal replacement therapy. Hematologic parameters showed severe leukopenia (0.74 × 10^9^/L), thrombocytopenia (72 × 10^9^/L), and elevated serum levels of procalcitonin and C-reactive protein (271.57 ng/ml and 43.41 mg/dl, respectively). Urine, blood cultures, and bronchoalveolar lavage (BAL) samples were collected immediately and forwarded to the hospital's microbiology service for urine antigen testing (UAT) and BAL sample culture to accurately diagnose *Legionella* infection.

The patient remained in the ICU for 7 days and was discharged to the ward, awake, without any kind of organ support, and maintaining only therapy against *Lp* and *Bb*.

### FilmArray analysis, culture examination, MALDI Biotyper identification, and serological assay

The BioFire FilmArray Pneumonia plus Panel (FAPP, Biomerieux), a multiplex PCR technique based on a syndromic approach, was used (11). This test can simultaneously identify 27 of the most common pathogens involved in lower respiratory tract infections (semi-quantitative results for 11 Gram-negative and 4 Gram-positive bacteria and qualitative results for 3 atypical bacteria and 9 viruses) and 7 antibiotic resistance genes. The BAL culture was performed on buffered charcoal yeast extract (BCYE, ThermoFisher, United Kingdom) and MacConkey agar plates media (ThermoFisher, United Kingdom) for *Lp* and Bb, respectively, and both were checked every 2 days. Suspected colonies were tested using a latex agglutination test.

Water samples from the water system of the hotel where the patient had stayed and from his home were collected and analyzed by culture according to ISO 11731.

Quantification of IgG and IgM against *Lp* was performed with anti-*Legionella pneumophila* (IgG–IgM) ELISA (Euroimmun, Germany). The results were interpreted and calculated according to manufacturers' instructions.

Furthermore, the isolated colonies of *Bb* were pre-treated using the ethanol/formic acid extraction procedure, as previously described (12), while MALDI Biotyper software, version 3.1 (Bruker Daltonics, Bremen, Germany), was used to process the raw spectra and to compare them for strain classification.

### Whole genome sequencing and typing

*Lp* serogroup 1 (*Lp*1), isolated from the patient's BAL, was analyzed by whole genome sequencing using Illumina technology. To this end, sequencing libraries were prepared using the Nextera XT DNA Library Preparation Kit (Illumina) and then run on two different Illumina platforms: a 150-bp paired-end sequencing run was performed on the NextSeq 500 (Illumina) using a Mid Output Kit v2 and a 250-bp paired-end sequencing run was performed on the MiSeq (Illumina) (BioProject: PRJNA1126987).

Core genome multi-locus sequence typing (cgMLST) was carried out on 46 ST1, including the patient's and other *Lp*1 strains isolated in France and Italy. The cgMLST scheme, based on 1,521 core genes (13), was converted using chewBBACA software (Galaxy version 2; 32), and a minimum spanning tree was visualized using GrapeTree software (14). The *Lp*1 clinical isolate was further typed using sequence-based typing and monoclonal antibody typing (15–17).

### Antimicrobial susceptibility test

The *Bp* colonies were analyzed for antimicrobial susceptibility according to the Clinical and Laboratory Standards Institute (CLSI). ETEST (bioMérieux, France) for *Bb* was performed on Mueller–Hinton agar with a 0.5 McFarland standard, using the following antimicrobials: penicillin G, ampicillin, cefotaxime, amikacin, tobramycin, nalidixic acid, trimethoprim-sulfamethoxazole, rifampicin, amoxicillin-clavulanate, moxifloxacin, imipenem, and meropenem. Minimum inhibitory concentration (MIC) values were determined after incubation at 37°C for 18–24 h.

The following eight antibiotics were tested against the *Lp* clinical strain using broth microdilution: azithromycin (0.015–8 mg/ml), clarithromycin, ciprofloxacin, levofloxacin, moxifloxacin (0.0009–0.5 μg/ml), erythromycin and doxycycline (0.03–16 μg/ml), and rifampicin (0.00005–0.03 μg/ml), as reported in the European Committee on Antimicrobial Susceptibility Testing (EUCAST) guidance document. For *Bp* susceptibility, tests were performed according to CLSI standards (18, 19).

## Results

### FilmArray and culture examination

*Lp* DNA was detected using the FilmArray method shortly after admission to the ICU. This enabled the ICU physicians to initiate targeted therapy with IV levofloxacin (750 mg) and empirical IV piperacillin/tazobactam (a 9 g loading dose followed by a continuous infusion for a total of 18 g per day was stopped on the 5^th^ day). After 4 days of incubation, the BAL culture provided the isolation of *Lp* serogroup 1 (*Lp*1). The UAT was positive and remained positive even after 7 months. In addition, the IgG and IgM titers (IgG = 0.3 U/ml; IgM = 2.7 U/ml) were positive.

The culture of the water samples collected from the water system of the hotel where the patient stayed did not provide any isolate, while the culture of the water samples from the patient's home provided *Lp* non-serogroup 1 at 3.5 × 10^3^-6.1 × 10^2^ CFU/L.

Interestingly, the microbiological culture of the BAL on selective MacConkey agar revealed *Bb*-positive colonies, identified using MALDI-TOF MS.

### Typing and WGS analysis

**Figure 1:**
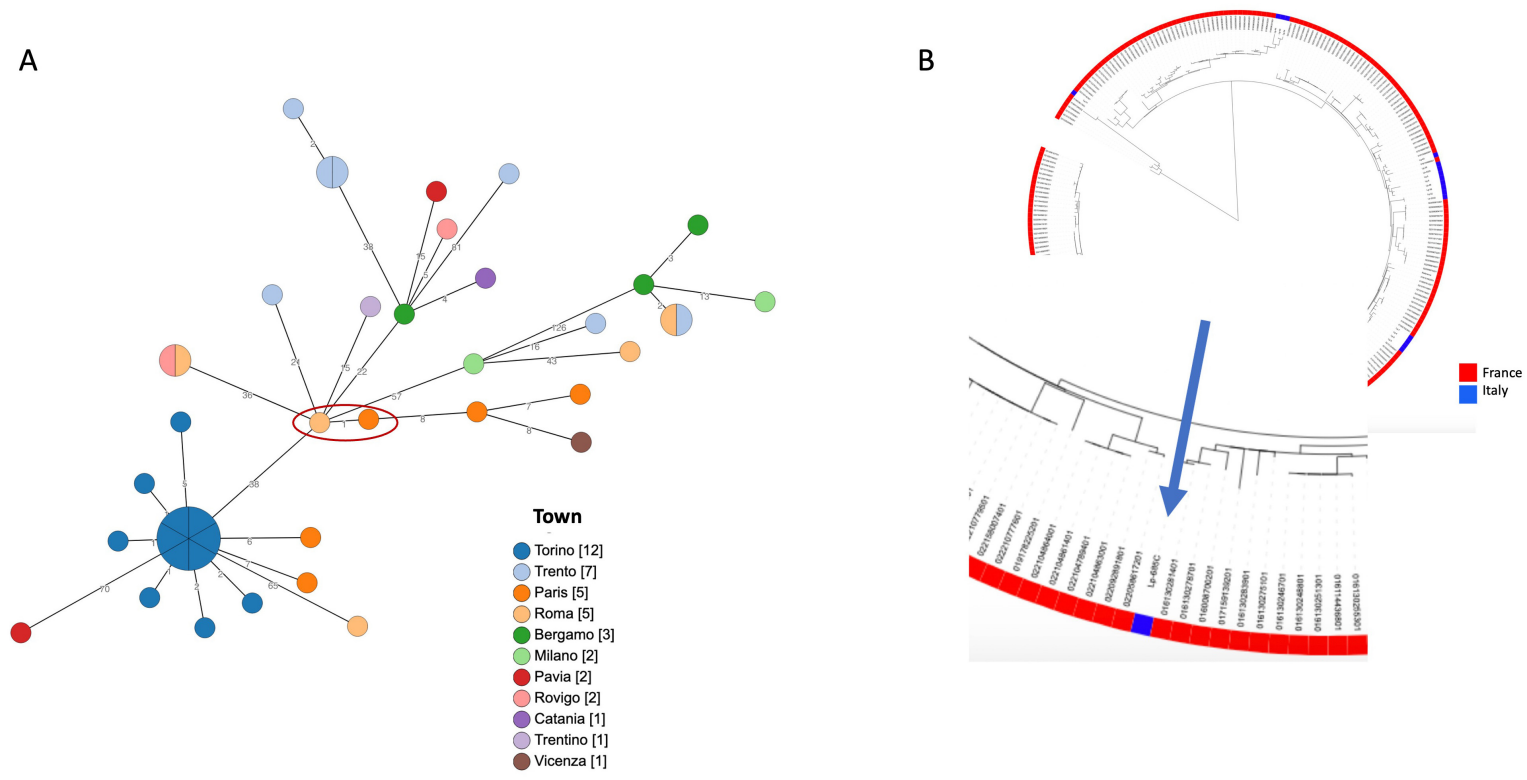
Phylogenetic analysis of ST1. **(A)** cgMLST based on the subset 1,471 cgMLST targets of the 1,521 core gene scheme is shown. Patient's ST1 and the most phylogenetically related strain are highlighted by a red circle. Node colors correspond to the town where strains were isolated, and on the branches the number of different loci is reported. In **(B)**, a maximum likelihood tree of a large number of ST1 genomes, available in GenBank, (in red those isolated in France, in blue those from Italy) is shown. The zoom on the patient's ST1 is highlighted by the arrow.

The *Lp*1 clinical isolate was typed as the Philadelphia monoclonal subgroup, ST1. cgMLST was produced and the gene profiles were visualized using a minimum spanning tree (Figure 1A). When the patient's ST1 was compared with other unrelated ST1 strains isolated in France and Italy, very similar gene profiles, ranging from 1 to 126 different loci, were found. In particular, an environmental ST1 strain isolated in a French hospital in 2019 showed a single locus of difference compared to the ST1 isolated from the patient (Figure 1A, red circle). As shown in Figure 1B, when the comparison was extended to a large number of ST1 genomes isolated in France and Italy, the phylogenetic analysis revealed that all these genomes are quite similar.

### Antimicrobial susceptibility test

MIC values determined for *Bb* and *Lp* are shown in Table 1.

**Table 1:** MIC values obtained for *B. bronchiseptica* and L. *pneumophila*.

***B. bronchiseptica***		***L. pneumophila***	
**Antimicrobial**	**MIC (**μ**g/ml)**	**Antimicrobial**	**MIC (mg/L)**
Penicillin G	>128	Azithromycin	0.5
Ampicillin	>128	Clarithromycin	0.06
Cefotaxime	>128	Erythromycin	0.5
Amikacin	32	Ciprofloxacin	0.03
Tobramycin	8	Levofloxacin	0.015
Nilidixic acid	32	Moxifloxacin	0.06
Trimethoprim-sulfamethoxazole	64	Rifampicin	0.0009
Rifampicin	128	Doxycycline	8
Amoxicillin-clavulanate	32/16		
Moxifloxacin	1		
Imipenem	4		
Meropenem	0.06		

## Discussion

To the best of our knowledge, we report the first case of *Lp*-*Bb* coinfection in an immunocompromised patient. Concomitants or sequential coinfections of *Lp* with other pathogens have rarely been reported, primarily involving viral and few bacterial coinfections, but never with *Bb* (20–24). *Bb* is primarily a zoonotic organism that is rarely encountered as a pathogen in cases of acute respiratory infections, which mainly involve immunocompromised people (10, 25). Although it was not possible to isolate *Bb* from the patient's dog, the regular contact between the patient and his dog (presumably with its upper respiratory tract colonized by *Bb*) likely allowed the transmission of the infection, exacerbated by the patient's immunosuppressive conditions. Although vaccination against kennel cough is a common practice in domestic pets, there is no official surveillance for *Bb* that could alert to the emergence of resistant strains (26). In addition, in the absence of reference methods and breakpoints from the CLSI or EUCAST for this organism, the results of susceptibility testing are difficult to interpret from a clinical point of view. No specific guidelines for the treatment of *Bb* infection are available. Furthermore, only a few specific studies have been published on the mechanisms of resistance in *Bb* clinical isolates (26). *Bb* has evolved a species-specific β-lactamase of the BOR-1 class, which could explain the highest level of penicillin MICs. Efflux mechanisms and/or reduced membrane permeability may also be related to the highest level of MICs for other β-lactams and cephalosporins (27).

Similarly, for Lp, reference protocols, breakpoints, and Epidemiological cut-off (ECOFF) values have not yet been established. However, based on the data reported in the literature, the isolated strain appears sensitive to all the antibiotics tested; even for azithromycin, it shows an MIC value considered on average higher than the value observed for most strains (28). *Lp*1 ST1 is globally spread in clinical cases and has been reported as a leading cause of community- and hospital-acquired LD in many countries (29). It is frequently found in the water systems of various buildings. New insights into the ST1 population genome structure, prone to recombination events that are at the basis of genetic diversity, revealed a wide divergence within this ST (30). For all these reasons, molecular epidemiological investigation requires not only genome matching between clinical and environmental strains but also an accurate analysis of ST1 genomes. Unfortunately, in this case, no environmental ST1 strain was isolated from the hotel where the patient stayed during the 10 days before the onset of the symptoms and where he presumably contracted the infection. The phylogenetic analysis highlighted a high similarity between the ST1 strains isolated in France and the patient's ST1, as well as with other unrelated ST1 strains isolated in Italy. Therefore, no correlation with the source of infection could be established. In this LD case, only the availability of both clinical and environmental strains, possibly in comparison with other unrelated ST1 genomes, can clarify the true source of infection.

Prompt molecular tests allowed for the initiation of the specific antibiotic therapy, which was fundamental in improving the patient's condition. Fortunately, no increased resistance to macrolides and fluoroquinolones was found for Lp1, although a possible increase has been reported recently. In addition, ascertaining sensitivity to all the antibiotics suggested by the EUCAST for *Bb* allowed the administration of the correct therapy for an immunocompromised patient (31, 32).

This case report focused on the following: (a) *Bb* as an opportunistic pathogen, particularly in patients with a previous history of respiratory disease and in those who are immunocompromised. In these patients, susceptibility testing should be routinely performed. Interpretative criteria for the clinical interpretation of MICs should be developed. (b) *Lp* is responsible for serious pneumonia, and the risk of contracting additional infections can further aggravate the patient's condition, making the outcome of the disease much more serious or even fatal. Based on the literature, no relationship can be established between this type of coinfection and the severity of the patient's disease. However, we can empirically assume that the patient likely experiences some degree of immunosuppression due to his hematological disorder (multiple myeloma with multiple relapses, treated with multiple cycles of chemotherapy), making him susceptible to both infections. For this reason, it is important to maintain a high suspicion of other possible infections, especially in elderly people and immunocompromised patients, by conducting as broad a screening as possible for other pathogens using rapid and sensitive nucleic acid amplification tests. This allows for targeted therapy and promotes better control against the infection caused by Lp, with a consequent reduction in severe sequela, hospital stays, and costs for public spending.

## Data Availability

The datasets presented in this study can be found in online repositories. The names of the repository/repositories and accession number(s) can be found in the article/supplementary material.
